# Supplementary material for: Framework to perform taint analysis and security assessment of IoT devices in smart cities
Source: PeerJ Comput Sci. 2023 Dec 21;9:e1771. doi: 10.7717/peerj-cs.1771 (PMC10773924; doi:10.7717/peerj-cs.1771)
Supplement: Supplemental Information 1 [file peerj-cs-09-1771-s001.docx]

Pseudo code 1: analyze IoT Firmware

| # Extract firmware image from IoT device  *firmware_image = extract_firmware(device)*  # Analyze firmware image  *analyze_result = analyze_firmware(firmware_image)*  # Perform firmware reverse engineering  *reverse_engineer_result = reverse_engineer_firmware(firmware_image,analyze_result)*  # Modify firmware  *patched_firmware = patch_firmware(firmware_image, custom_payloads)*  # Test firmware and bootloader  *test_results = test_firmware(patched_firmware)*  # Generate report  *generate_report(analyze_result,reverse_engineer_result, test_results)* |
| --- |
