# Supplementary material for: Framework to perform taint analysis and security assessment of IoT devices in smart cities
Source: PeerJ Comput Sci. 2023 Dec 21;9:e1771. doi: 10.7717/peerj-cs.1771 (PMC10773924; doi:10.7717/peerj-cs.1771)
Supplement: Supplemental Information 2 [file peerj-cs-09-1771-s002.docx]

| *while (1)*  *{*  *// Check for firmware update command*  *if (CheckForUpdateCommand())*  *{*  *// Erase old firmware from memory*  *EraseFirmware();*  *// Receive new firmware over UART*  *ReceiveFirmwareOverUART();*  *// Verify the firmware*  *if (VerifyFirmware()) {*  *// If firmware is verified, jump to new firmware*  *JumpToNewFirmware();*  *} else {*  *// If firmware is not verified, stay in the bootloader*  *SendErrorOverUART("Firmware verification failed");*  *}*  *}*  *}* |
| --- |

Pseudo Code 2: Firmware update
