# Supplementary material for: Framework to perform taint analysis and security assessment of IoT devices in smart cities
Source: PeerJ Comput Sci. 2023 Dec 21;9:e1771. doi: 10.7717/peerj-cs.1771 (PMC10773924; doi:10.7717/peerj-cs.1771)
Supplement: Supplemental Information 3 [file peerj-cs-09-1771-s003.docx]

Pseudo Code 3: Bootloader Security Assessment

| /*/ Perform memory overflow test*  *if (TestMemoryOverflow())*  *{*  *// Handle memory overflow error*  *SendErrorOverUART("Memory overflow detected");*  *}*  *// Perform firmware integrity check*  *if (!VerifyFirmwareIntegrity())*  *{*  *// Handle firmware integrity error*  *SendErrorOverUART("Firmware integrity check failed");*  *}*  *// Perform firmware update authenticity check*  *if (!VerifyFirmwareUpdateAuthenticity())*  *{*  *// Handle firmware update authenticity error*  *SendErrorOverUART("Firmware update authenticity check failed");*  *}*  *// Perform secure boot check*  *if (!VerifySecureBoot())*  *{*  *// Handle secure boot error*  *SendErrorOverUART("Secure boot check failed");*  *}*  *3*  *// Perform code signing check*  *if (!VerifyCodeSigning())*  {  // Handle code signing error  SendErrorOverUART("Code signing check failed");  } |
| --- |
