# Supplementary material for: Framework to perform taint analysis and security assessment of IoT devices in smart cities
Source: PeerJ Comput Sci. 2023 Dec 21;9:e1771. doi: 10.7717/peerj-cs.1771 (PMC10773924; doi:10.7717/peerj-cs.1771)
Supplement: Supplemental Information 4 [file peerj-cs-09-1771-s004.docx]

Pseudo Code 4: Hardware Initialization code in a Bootloader

| // Initialize memory  *InitializeRAM();*  // Initialize communication interfaces  *InitializeUART();*  *InitializeSPI();*  *InitializeI2C();*  // Initialize peripherals  *InitializeGPIO();*  *InitializeADC();*  *InitializeDAC();*  // Perform basic hardware tests  *if (!TestRAM())*  {  // Handle RAM error  }  *if (!TestUART())*  {  // Handle UART error  }  *if (!TestSPI())*  {  // Handle SPI error  }  *if (!TestI2C())*  {  // Handle error  }  *if (!TestGPIO())*  {  // Handle error  }  *if (!TestADC())*  {  // Handle ADC error  }  *if (!TestDAC())*  {  // Handle DAC error  }  // If all tests = pass, then jump to the main firmware  *Jump-To-Main-Firmware();* |
| --- |
