# Supplementary material for: Framework to perform taint analysis and security assessment of IoT devices in smart cities
Source: PeerJ Comput Sci. 2023 Dec 21;9:e1771. doi: 10.7717/peerj-cs.1771 (PMC10773924; doi:10.7717/peerj-cs.1771)
Supplement: Supplemental Information 5 [file peerj-cs-09-1771-s005.docx]

**Research Paper: Framework to Perform Taint Analysis and Security Assessment of IoT Devices**

Taint analysis is a technique used to track the flow of tainted or untrusted data through a program or system. In the context of IoT devices, taint analysis can be used to identify potential security vulnerabilities or data leakage by tracing how sensitive information flows through the device's software. We have provided the pseudo-code representation of a simple taint analysis algorithm for an IoT device.

Code details:

| function markTainted(source, data)  // Mark the data as tainted and associate it with the source  data.tainted = true  data.taintSource = source  function propagateTaint(srcData, destData)  // Propagate taint from srcData to destData  if srcData.tainted  destData.tainted = true  destData.taintSource = srcData.taintSource  function analyzeIoTDevice(device)  for each component in device.components  for each input in component.inputs  // Assume inputs come from external sources (sensors, network, etc.)  markTainted("External Input", input)  // Process components and propagate taint  for each component in device.components  for each instruction in component.instructions  if instruction.usesTaintedData  propagateTaint(instruction.sourceData, instruction.destData)  // Analyze and report tainted outputs  for each component in device.components  for each output in component.outputs  if output.tainted  report("Tainted Data Leakage Detected: Output", output, output.taintSource)  // Main entry point  function main()  device = initializeDevice()  analyzeIoTDevice(device) |
| --- |

Tain Analysis – SQL Injection

| using System.Reflection;  using System.Runtime.InteropServices;  // General Information about an assembly is controlled through the following  // set of attributes. Change these attribute values to modify the information  // associated with an assembly.  [assembly: AssemblyTitle("Model")]  [assembly: AssemblyDescription("")]  [assembly: AssemblyConfiguration("")]  [assembly: AssemblyCompany("")]  [assembly: AssemblyProduct("Model")]  [assembly: AssemblyCopyright("Copyright © 2022")]  [assembly: AssemblyTrademark("")]  [assembly: AssemblyCulture("")]  // Setting ComVisible to false makes the types in this assembly not visible  // to COM components. If you need to access a type in this assembly from  // COM, set the ComVisible attribute to true on that type.  [assembly: ComVisible(false)]  // The following GUID is for the ID of the typelib if this project is exposed to COM  [assembly: Guid("536b46b7-7816-4273-932f-11cbe4155d38")]  // Version information for an assembly consists of the following four values:  //  // Major Version  // Minor Version  // Build Number  // Revision  //  // You can specify all the values or you can default the Build and Revision Numbers  // by using the '*' as shown below:  // [assembly: AssemblyVersion("1.0.*")]  [assembly: AssemblyVersion("1.0.0.0")]  [assembly: AssemblyFileVersion("1.0.0.0")] |
| --- |

Source Area

| namespace Model.Rules  {  /// <summary>  /// </summary>  public class SourceArea  {  /// <summary>  /// Gets or sets the label.  /// </summary>  /// <value>The label.</value>  public string Label { get; set; }  /// <summary>  /// Gets or sets the path.  /// </summary>  /// <value>The path.</value>  public string Path { get; set; }  }  } |
| --- |

Taint Propagation Rules:

| using System.Collections.Generic;  namespace Model.Rules  {  /// <summary>  /// Model.Rules <c>TaintPropagationRules</c> class.  /// <para>  /// Contains all the information gained from the .json config file for  /// solving the taint variable propagation problems. Each taint variable  /// propagation problem will be solved with the consideration of these  /// rules, therefore by changing these rules it is possible to manipulate  /// with the final result and adjust it according to our needs.  /// </para>  /// </summary>  public class TaintPropagationRules  {  /// <summary>  /// Maximal level of recursion in method-body blocks during  /// interprocedural analysis. If the Scope is not interprocedural, then  /// the Level will not be taken into consideration during analysis.  /// </summary>  /// <value>integer - max level of recursion during interp. analysis.  /// </value>  public int Level { get; set; }  /// <summary>  /// Gets or sets the source areas used to label the results for easier  /// orientation in results.  /// </summary>  /// <value>The list of source areas.</value>  public List<SourceArea> SourceAreas { get; set; }  /// <summary>  /// Gets or sets the list of sink methods. Sink methods are methods  /// which are considered as potentialy vulnerable if at least one  /// unchecked user-provided argument is passed to them. For example  /// methods which communicate with a database.  /// </summary>  /// <value>The list of sink methods.</value>  public List<string> SinkMethods { get; set; }  /// <summary>  /// Gets or sets the list of cleaning methods. Cleaning methods are  /// methods which are considered as safe under any circumstances, and at  /// any time, any argument can be passed to them without the need to  /// track the argument. It is immediately considered as cleaned and  /// tracking the argument may be considered as finished.  /// </summary>  /// <value>The list of cleaning methods.</value>  public List<string> CleaningMethods { get; set; }  }  } |
| --- |

Solution Scan Results Module:

| using System;  using System.Collections.Generic;  using Model.CSProject;  namespace Model.Solution  {  /// <summary>  /// Model.Solution <c>SolutionScanResult</c> class.  /// <para>  /// Contains all the information gained during the analysis of the specific  /// .sln file.  /// </para>  /// </summary>  public class SolutionScanResult  {  public DateTime SolutionScanResultStartTime { get; set; }  public DateTime SolutionScanResultEndTime { get; set; }  public TimeSpan SolutionScanResultTotalTime  { get { return SolutionScanResultEndTime - SolutionScanResultStartTime; } }  /// <summary>  /// Gets or sets the path of the analysed .sln file.  /// </summary>  public string Path { get; set; }  /// <summary>  /// Gets or sets the list of the separate csproj scan results. Every  /// single dependency mentioned in the .sln has its own results stored  /// here. The result of the analysis of csproj file is stored as a  /// <see cref="CSProjectScanResult"/>.  /// </summary>  public List<CSProjectScanResult> CSProjectScanResults { get; set; } = new List<CSProjectScanResult>();  public List<String> PathsOfSkippedCSProjects { get; set; } = new List<String>();  /// <summary>  /// Gets or sets the number of all .csproj files under the analysed  /// solution.  /// </summary>  /// <value>The number of all .csproj files.</value>  public int NumberOfCSProjFiles { get; set; } = 0;  }  } |
| --- |
